# BRASH Phenomenon: A Case Report on the Dangerous Combination of Bradycardia, Acute Renal Failure, Atrioventricular Nodal Blockade, Shock, and Hyperkalemia

**DOI:** 10.7759/cureus.90898

**Published:** 2025-08-24

**Authors:** Tetyana Okan, Azeem Arastu, Mehrdad Zarghami, Vidhi Patel, Uchenna Chinakwe, Zoran Lasic

**Affiliations:** 1 Internal Medicine, Jamaica Hospital Medical Center, New York, USA; 2 Cardiology, Lenox Hill Hospital, New York, USA

**Keywords:** acute renal failure, atrioventricular nodal blockers, bradycardia, brash syndrome, cardiogenic shock, diagnosis of brash syndrome, hemodialysis, hyperkalemia, management of brash syndrome, shock

## Abstract

Bradycardia, renal failure, AV nodal blockade, shock, and hyperkalemia (BRASH) syndrome is a rare medical phenomenon that includes the aforementioned symptoms. It can lead to multisystem organ failure, resulting in high mortality.

We are reporting a case of BRASH syndrome in a 70-year-old female with a history of chronic kidney disease, severe rheumatic mitral stenosis and paroxysmal atrial fibrillation who presented to the emergency department after an episode of presyncope and worsening shortness of breath. The patient’s home medications included carvedilol and apixaban (due to non-compliance with warfarin). Her initial blood pressure (BP) was 162/99 mmHg and heart rate (HR) 28 beats/min. EKG showed junctional bradycardia. After administration of atropine for symptomatic bradycardia and glucagon to reverse beta-blocker activity given poor response to initial atropine treatment, there was a transient improvement of HR to 43 beats/min. However, her BP decreased to 70/40 mmHg, requiring dopamine infusion.

Laboratory findings showed significantly elevated blood urea nitrogen (BUN) at 114 mg/dL, creatinine at 12.4 mg/dL (baseline creatinine 6 mg/dL), and critical hyperkalemia of 7.9 mEq/L. These results prompted immediate treatment with intravenous calcium gluconate and an insulin-dextrose infusion, along with oral sodium zirconium cyclosilicate and albuterol nebulization.

The patient was admitted to the intensive care unit for management of cardiogenic shock with refractory bradycardia and acute kidney injury with severe hyperkalemia requiring hemodialysis.

She underwent urgent hemodialysis with resolution of hyperkalemia and improvement of HR to 68 beats/min and rhythm to sinus, thus, there was no need for pacemaker placement. She was discharged home with initiation of outpatient dialysis.

Early recognition of BRASH syndrome is crucial because its management differs significantly from the standard Advanced Cardiovascular Life Support (ACLS) bradycardia algorithm. Unlike the standard approach, therapy for BRASH syndrome extends beyond atropine and electrolyte correction to potentially include hemodynamic vasopressor support, temporary transcutaneous pacing, and urgent renal replacement therapy.

## Introduction

Bradycardia, renal failure, AV nodal blockade, shock, and hyperkalemia (BRASH) syndrome is a rare medical phenomenon that can lead to multisystem organ failure. It has a prevalence of 0.04% [1] in the emergency department setting and an inpatient mortality of 5.7% [2,3]. The pathophysiology of BRASH phenomenon remains unclear; however, it is likely related to impaired renal clearance of AV-nodal blocking agents, in particular beta-blockers with renal elimination. Thus, a synergistic effect between AV-nodal blockers and hyperkalemia exacerbates bradycardia [1,2]. Commonly used agents associated with BRASH syndrome include beta-blockers, calcium channel blockers, amiodarone and digoxin. Bradycardia in BRASH syndrome is profound and persistent, which is out of proportion to the degree of hyperkalemia. It subsequently leads to reduced cardiac output and cardiogenic shock with worsening renal perfusion, which subsequently further propagate hyperkalemia. This vicious pathophysiological cycle is classified as BRASH syndrome [1,2]. 

BRASH phenomenon is more frequently observed in older patients with chronic cardiac and renal pathology. BRASH syndrome is commonly triggered by hypovolemia, sepsis, dehydration, and certain medications such as ranolazine (due to its bradycardic effect) and sulfamethoxazole/trimethoprim (due to reducing renal potassium excretion leading to potassium retention) [1,4]. 

## Case presentation

A 70-year-old female with a history of chronic kidney disease, hypertension, rheumatic mitral stenosis and paroxysmal atrial fibrillation presented to the emergency department (ED) after an episode of presyncope and worsening shortness of breath. Her home therapy included carvedilol 6.25 mg twice daily, nifedipine 90 mg daily, ramipril 10 mg, hydrochlorothiazide 25 mg daily and apixaban 5 mg twice daily. Apixaban was initiated due to her noncompliance with warfarin therapy. Worsening kidney function was noticed for the last two years with a baseline creatinine of 6 mg/dL. As per Emergency Medical Services, she was found to be in respiratory distress and bradycardic with a heart rate (HR) of 28 beats/min; thus, she was placed on a non-rebreather mask and atropine 1 mg was administered.

**Figure 1:**
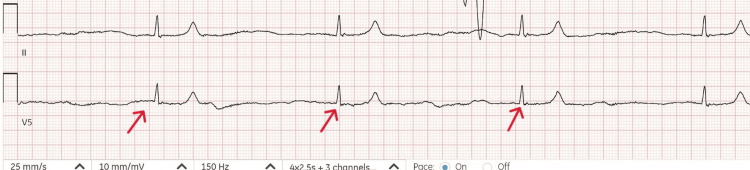
EKG revealed junctional bradycardia with ventricular rate of 24 beats/min (arrows).

On arrival to the ED, the patient's blood pressure (BP) was 162/99 mmHg and her HR slowed from 43 to 24 beats/minute. Electrocardiogram (ECG) showed junctional bradycardia with a ventricular rate of 24 beats/min (Figure 1). Physical examination revealed a diastolic murmur and 3+ pitting edema in the lower extremities. 

Initial laboratory results were remarkable for severe hyperkalemia (7.9 mEq/L), blood urea nitrogen (BUN) 114 mg/dL, creatinine 12.4 mg/dL, non-anion gap metabolic acidosis and elevated B-type natriuretic peptide (BNP) 28,700 (Table 1). 

**Table 1:** Initial laboratory results showing acute kidney injury with creatinine 12.4 mg/dL, severe hyperkalemia 7.9 mEq/L, and non-anion gap metabolic acidosis with bicarbonate of 18 mEq/L.

Chemistry	Reference Range and Units	Patient’s labs
Glucose	74 - 106 mg/dL	172 mg/dL
Urea Nitrogen	7 - 17 mg/dL	114 mg/dL
Creatinine	0.5 - 1.0 mg/dL	12.4 mg/dL
Sodium	137 - 145 mEq/L	126 mEq/L
Potassium	3.5 - 5.1 mEq/L	7.9 mEq/L
Chloride	98 - 107 mEq/L	96 mEq/L
Carbon Dioxide	22 - 30 mEq/L	18 mEq/L
Calcium	8.4 - 10.2 mg/dL	7.5 mg/dL
Anion Gap	5.00 - 16.00 mEq/L	12.00 mEq/L

**Figure 2:**
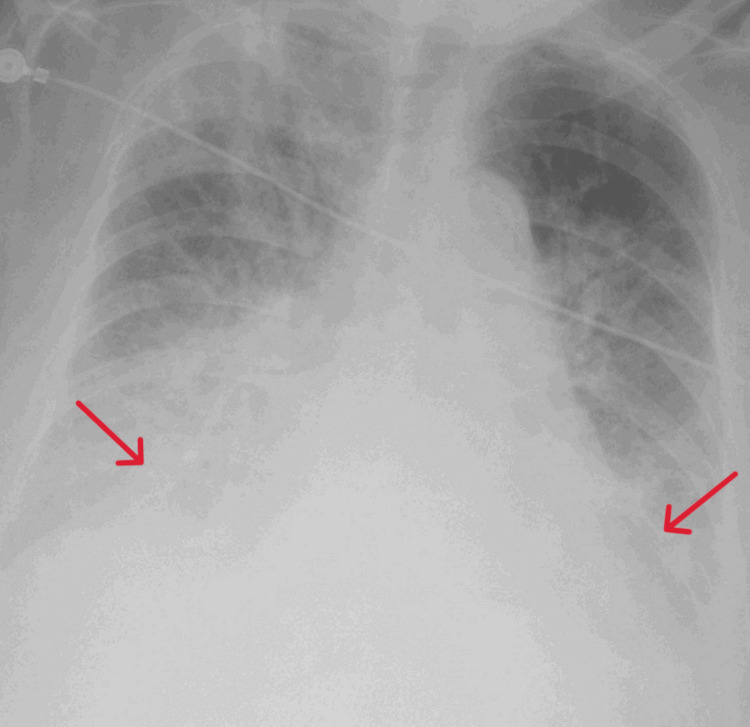
Chest X-ray demonstrated a prominent bilateral pulmonary vasculature and bilateral pleural effusions (arrows).

Chest radiography showed bilateral congestion with bilateral pleural effusions (Figure 2).

**Figure 3:**
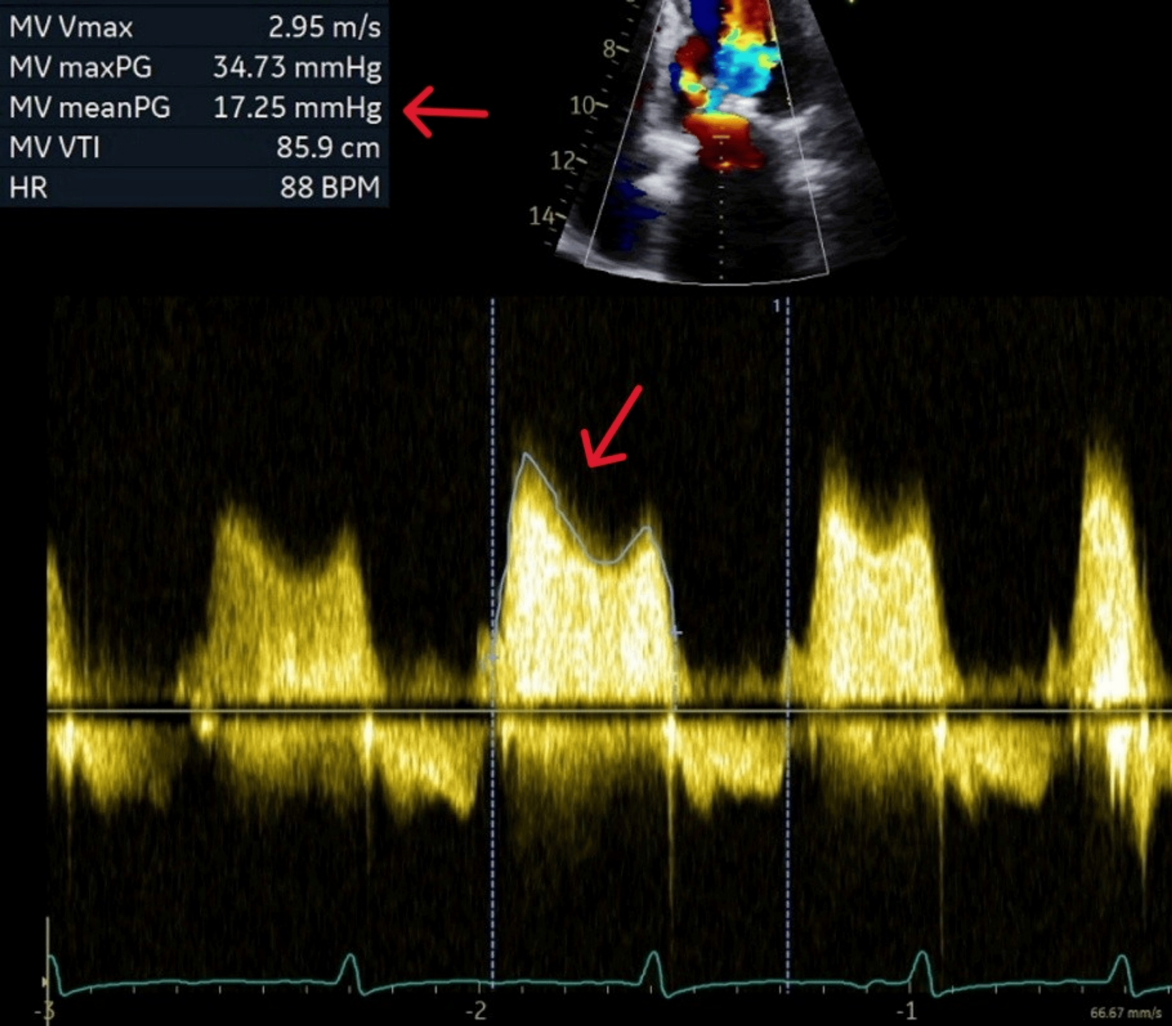
Transthoracic echocardiography, continuous wave doppler showing high mean pressure gradient (arrows).

**Figure 4:**
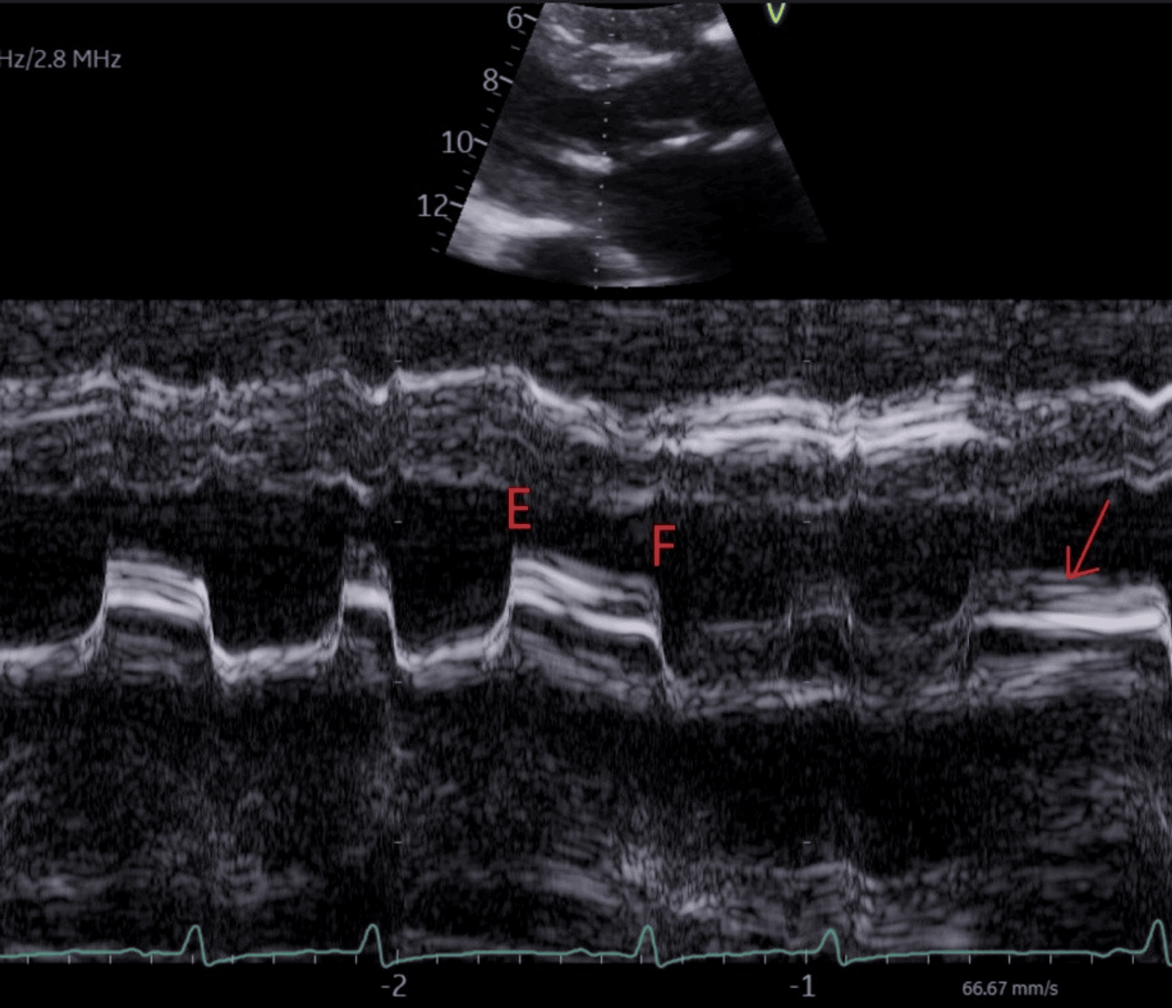
Transthoracic echocardiography, M-mode, showing thickened mitral valve leaflets (arrow) with reduced E-F slope of the anterior mitral leaflet and absent mitral valve A-wave.

Transthoracic echocardiography was suggestive of chronic rheumatic heart disease with severe thickening of both mitral valve leaflets causing moderate to severe stenosis with mitral valve mean gradient of 17.25 mmHg, calculated mitral valve area of 1.2 cm2, left ventricular ejection fraction of 50% and moderate pulmonary hypertension (Videos 1, 2, Figures 3, 4).

**Video 1:** Mitral stenosis, parasternal long axis view. Rheumatic heart disease with severe thickening of both mitral valve leaflets, left atrial enlargement, and left ventricular ejection fraction (LVEF) of 50%.

**Video 2:** Mitral stenosis, parasternal short axis view. Severe thickening of both mitral valve leaflets and profound calcinosis of posterior mitral leaflet.

**Figure 5:**
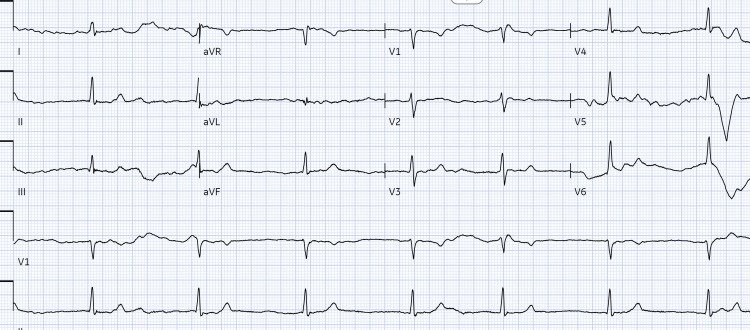
Repeat EKG after therapy with calcium gluconate, sodium zirconium cyclosilicate and insulin-dextrose therapy showing transient and mild improvement of ventricular rate to 43 beats/min.

Emergency interventions included atropine for symptomatic bradycardia followed by glucagon administration for beta-blocker reversal given poor response to initial atropine treatment. The patient was placed on bilevel positive airway pressure (BiPAP) and diuresed with furosemide. Calcium gluconate, sodium zirconium cyclosilicate, regular insulin and 50% dextrose in water were administered for hyperkalemia followed by albuterol nebulization. After receiving treatment for symptomatic bradycardia, the patient had a transient improvement in HR rate to 43 beats/minute (Figure 5).

However, she became hypotensive with BP decreasing to 70/40 mmHg, triggering pressor therapy. Dopamine infusion was chosen due to its chronotropic effect. She was admitted to the intensive care unit for management of cardiogenic shock, refractory bradycardia in the setting of beta-blocker use, and acute kidney injury with severe hyperkalemia requiring hemodialysis. Additional workup was done, which ruled out acute myocardial infarction, pulmonary embolism, and sepsis, narrowing the diagnosis to metabolic and medication-related causes. 

Urgent hemodialysis was initiated, which led to normalization of potassium level from 7.9 to 3.6 mEq/L and significant improvement of renal function: BUN from 114 to 52 mg/dL and creatinine from 12.4 to 6 mg/dL (Table 2). 

**Table 2:** Post hemodialysis laboratory results showing significant improvement of renal function (creatinine 6.0 mg/dL), resolution of hyperkalemia (K 3.6 mEq/L) and metabolic acidosis (bicarbonate 25 mEq/L).

Chemistry	Reference Range and Units	Patient’s labs
Glucose	74 - 106 mg/dL	92 mg/dL
Urea Nitrogen	7 - 17 mg/dL	52 mg/dL
Creatinine	0.5 - 1.0 mg/dL	6.0 mg/dL
Sodium	137 - 145 mEq/L	137 mEq/L
Potassium	3.5 - 5.1 mEq/L	3.6 mEq/L
Chloride	98 - 107 mEq/L	96 mEq/L
Carbon Dioxide	22 - 30 mEq/L	25 mEq/L
Calcium	8.4 - 10.2 mg/dL	8.8 mg/dL
Anion Gap	5.00 - 16.00 mEq/L	16.00

**Figure 6:**
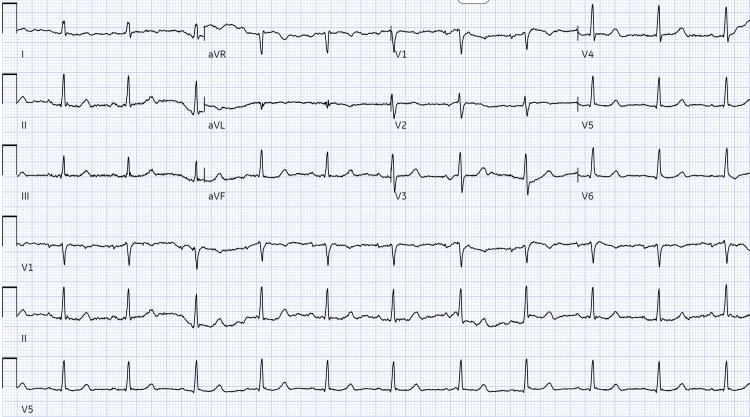
Post hemodialysis EKG showing a resolution of bradycardia and return to normal heart rate of 68 beats/min.

Junctional bradycardia resolved after the hemodialysis session. The rhythm was reverted to sinus with an improvement in heart rate to 68 beats/min as seen on repeat EKG (Figure 6), eliminating the need for pacemaker placement.

The patient remained asymptomatic with stable renal function and no recurrence of hyperkalemia or bradycardia. The decision was made to resume a beta-blocker on discharge for atrial fibrillation with rapid ventricular response. The patient was recommended to continue hemodialysis and was discharged home with the initiation of outpatient dialysis. She required 3 liters of oxygen via nasal cannula during ambulation with a rolling walker, thus, portable home oxygen was delivered. Due to functional decline, subacute rehab was recommended; however, the family declined this option.

## Discussion

BRASH syndrome represents a vicious cycle involving bradycardia, hyperkalemia, AV-nodal blockade, shock and renal failure. The pathophysiology involves impaired renal clearance, leading to the accumulation of AV-nodal blocking agents and potassium, which in turn exacerbates bradycardia and hyperkalemia. The BRASH syndrome can be triggered by dehydration, sepsis, or any cause of hypoperfusion or renal dysfunction [1,5]. Key diagnostic features include bradycardia that's out of proportion to the degree of hyperkalemia, alongside concurrent AV-nodal blockade and renal dysfunction, as observed in our case. Specific electrocardiographic changes, such as junctional rhythm, PR segment prolongation, and peaked T waves, were described in previously published case reports [2,4]. According to Majeed et al., 50% of patients with BRASH syndrome presented with junctional escape rhythm, while 17.1% had sinus bradycardia, and 12.9% complete heart block [2]. 

When evaluating a patient with bradycardia, hyperkalemia, and renal dysfunction, multiple conditions must be considered to differentiate BRASH syndrome from other common etiologies. BRASH phenomenon is distinct from simple acute hyperkalemia (due to acute renal failure or high potassium intake) because the latter doesn't involve the characteristic, cyclical relationship between AV-nodal blockade and bradycardia. In addition, isolated hyperkalemia generally will not precipitate bradycardia unless severe, usually more than 7 meq/L, while BRASH phenomenon is usually associated with moderate hyperkalemia [1,5-7]. Isolated AV-nodal blockade resulting from an overdose of beta-blockers, calcium channel blockers, or amiodarone is also in the differential diagnosis, especially when there's no concurrent renal impairment or hyperkalemia. These patients, however, typically respond better to atropine and glucagon for reversal. Digoxin toxicity, is additional medication related differential diagnosis which presents with bradycardia and hyperkalemia, however, it is often accompanied by characteristic ECG findings such as ST segment depression (“scooped” ST segments), shortened QT interval as well as other characteristic symptoms, such as nausea, vomiting, blurred vision, yellow-green discoloration of vision and confusion. Some endocrinopathies, including adrenal insufficiency, particularly Addison's crisis, can also mimic BRASH syndrome by causing bradycardia and hyperkalemia. Severe hypothermia could also present with bradycardia, electrolyte abnormalities, and renal dysfunction. 

Management of BRASH syndrome is challenging and requires a multidisciplinary approach. It focuses on interrupting the cycle of BRASH phenomenon with the following: 1) withdrawing AV-nodal blocking agents and reversal therapy with glucagon when patient is hemodynamically unstable; 2) hyperkalemia correction with calcium gluconate, insulin-dextrose therapy, sodium zirconium cyclosilicate [3,8]; 3) providing hemodynamic support and improving renal perfusion with vasopressors [1,3,8]; 4) temporary or permanent cardiac pacing [9]; and 5) initiating renal replacement therapy for refractory hyperkalemia [6,10]. 

According to published data, 32.9% of patients typically require cardiac pacing, and approximately 20% need renal replacement therapy [2,3,9,10]. In most cases, the lack of pacemaker dependence suggests BRASH syndrome is reversible with prompt management. 

Early recognition of BRASH syndrome and a multidisciplinary approach were critical in the management of our patient’s profound and persistent bradycardia. Collaboration among cardiology, nephrology, and critical care teams ensured early recognition and timely intervention. Our patient successfully recovered and was discharged in a stable condition with outpatient hemodialysis and a tailored medication regimen to avoid the recurrence of BRASH syndrome. Thorough medication review and dose adjustments of medications in renal impairment, as well as patient education, are crucial steps in preventing hospital readmissions. Long-term management strategies included regular follow-up with nephrology and cardiology teams and electrolyte monitoring for at least every two weeks during the initial recovery phase. Once electrolytes are stable, restarting beta-blockers should be a case-by-case decision, weighing risks and benefits due to a lack of standard guidelines and sufficient published data. In our case, the beta-blocker was resumed on discharge for atrial fibrillation with rapid ventricular response. 

The current data on BRASH syndrome is limited to published case reports. A retrospective analysis performed at the emergency department of the Buergerspital Solothurn in Switzerland between 2017 and 2018 analyzed 65,489 patient charts and confirmed only eight cases that met all criteria of BRASH syndrome [6]. More retrospective studies are needed to evaluate the prevalence of this syndrome, the rate of complications and the most effective therapeutic options for this rare phenomenon.

## Conclusions

This case highlights the importance of recognizing BRASH syndrome as a distinct clinical entity. It is characterized by persistent, profound bradycardia disproportionate to hyperkalemia levels. Early recognition and prompt therapeutic interventions are crucial to reduce its morbidity and mortality.

Further studies are needed to elucidate the syndrome’s pathophysiology and compare therapeutic options to establish standardized management protocols. 
